# Life-course influences of poverty on violence and homicide: 30-year Brazilian birth cohort study

**DOI:** 10.1093/ije/dyae103

**Published:** 2024-08-09

**Authors:** Joseph Murray, Michelle Degli Esposti, Christian Loret de Mola, Rafaela Martins, Andrew D A C Smith, Terrie E Moffitt, Jon Heron, Vanessa Iribarrem Miranda, Natalia Lima, Bernardo L Horta

**Affiliations:** Human Development and Violence Research Centre, Federal University of Pelotas, Pelotas, Brazil; Postgraduate Program in Epidemiology, Federal University of Pelotas, Pelotas, Brazil; Human Development and Violence Research Centre, Federal University of Pelotas, Pelotas, Brazil; Postgraduate Program in Epidemiology, Federal University of Pelotas, Pelotas, Brazil; Human Development and Violence Research Centre, Federal University of Pelotas, Pelotas, Brazil; Postgraduate Program in Public Health, Federal University of Rio Grande, Rio Grande, Brazil; Universidad Científica del Sur, Lima, Peru; Human Development and Violence Research Centre, Federal University of Pelotas, Pelotas, Brazil; Postgraduate Program in Epidemiology, Federal University of Pelotas, Pelotas, Brazil; Mathematics and Statistics Research Group, University of the West of England, Bristol, UK; Psychology and Neuroscience, Duke University, Durham, NC, USA; Psychiatry and Behavioral Sciences, and Center for Genome and Computational Biology, Duke School of Medicine, Duke University, Durham, NC, USA; Social, Genetic and Developmental Psychiatry Centre, Institute of Psychiatry, King’s College London, London, UK; Population Health Science, Bristol Medical School, Bristol, UK; Postgraduate Program in Epidemiology, Universidade do Extremo Sul Catarinense, Criciúma, Brazil; Postgraduate Program in Epidemiology, Federal University of Pelotas, Pelotas, Brazil; Postgraduate Program in Epidemiology, Federal University of Pelotas, Pelotas, Brazil

**Keywords:** Poverty, violence, homicide, birth cohort, Brazil

## Abstract

**Background:**

Homicide is the leading cause of death among young people in Latin America, one of the world’s most violent regions. Poverty is widely considered a key cause of violence, but theories suggest different effects of poverty, depending on when it is experienced in the life-course. Longitudinal studies of violence are scarce in Latin America, and very few prospective data are available worldwide to test different life-course influences on homicide.

**Methods:**

In a prospective birth cohort study following 5914 children born in southern Brazil, we examined the role of poverty at birth, in early childhood, and in early adulthood on violence and homicide perpetration, in criminal records up to age 30 years. A novel Structured Life Course Modelling Approach was used to test competing life-course hypotheses about ‘sensitive periods’, ‘accumulation of risk’, and ‘downward mobility’ regarding the influence of poverty on violence and homicide.

**Results:**

Cumulative poverty and poverty in early adulthood were the most important influences on violence and homicide perpetration. This supports the hypothesis that early adulthood is a sensitive period for the influence of poverty on lethal and non-lethal violence. Results were replicable using different definitions of poverty and an alternative outcome of self-reported fights.

**Conclusion:**

Cumulative poverty from childhood to adulthood was an important driver of violence and homicide in this population. However, poverty experienced in early adulthood was especially influential, suggesting the importance of proximal mechanisms for violence in this context, such as unemployment, organized crime, drug trafficking, and ineffective policing and justice systems.

Key Messages

- Interpersonal violence is the leading cause of death among young people in Latin America, but few longitudinal data are available about life-course determinants in that region.
- Poverty may have different effects on violence depending on when it is experienced in the life-course.
- The influence of poverty on violence and homicide was studied in a large cohort, including over five thousand children followed from birth to age 30 years in Southern Brazil.
- Cumulative poverty from childhood to adulthood was an important influence on violence and homicide, but poverty experienced in young adulthood had the largest effect.

## Introduction

Homicide caused over four times as many deaths as war and terrorism combined in the first two decades of the 21^st^ century, with the highest rates found in Central and Latin America and sub-Saharan Africa.^1^ In Brazil, 1 096 000 people have died from homicide since the turn of the century,^2^ and interpersonal violence is now *the* leading cause of death among young people.^1^^,^^3^ Longitudinal studies are important to discover prospective determinants of violence, and sensitive periods of exposure for prevention.^4^^,^^5^ Although important social, relationship, and individual risk factors for violence have been documented in longitudinal research in high-income countries,^6^^,^^7^ considerably less evidence is available in low- and middle-income countries (LMICs) where the highest rates of violence are found.

Two previous Brazilian longitudinal studies highlighted poverty as a key risk factor for crime and violence.^8^^,^^9^ Homicide specifically has also been linked to poverty in retrospective surveys, and small studies of children exposed to abuse,^10^ but to our knowledge only one prior study worldwide has examined prospective risk factors for homicide in a community sample, in Pittsburgh, USA. In that study including 39 homicide offenders,^11^ socioeconomic status at age 13 years showed an association with homicide that was positive but not significant.

Social epidemiology highlights multiple mechanisms linking inequality and poverty to health and behaviour, including material hardship, psychosocial stress, and macro-level policies undermining services in poorer populations.^12–14^ In criminology, Merton’s strain theory proposed that failure to attain wealth via legal means increases social strain, motivating use of crime.^15^^,^^16^ Rational choice theory,^17^ as well as strain theory, focuses on the effects of current socioeconomic conditions, suggesting that poverty primarily influences crime from adolescence onwards. Other theories emphasize the importance of early-life poverty for later crime, such as Moffitt’s theory^18^^,^^19^ of life-course persistent offending. In Sampson and Laub’s theory,^20^ poverty contributes to experiencing a ‘conveyor belt’ of disadvantage and rejection by prosocial institutions in a pathway to crime,^21^ and accumulation of poverty through time seems particularly important to this theory.

Important theories suggest different life-course periods when poverty is most important for crime and violence, but three gaps in knowledge stand out. First, there is little longitudinal research in societies with the highest rates of violence. Second, to our knowledge, only one community survey worldwide has examined prospective risk factors for homicide. Third, we located no prior study testing different life-course hypotheses about the influence of poverty on violence or homicide.

We investigated the following research questions in a Brazilian setting. First, does **accumulation** of poverty through the life-course increase risk for violence/homicide? Second, does experiencing poverty during a **sensitive period** in the life-course (e.g. early childhood) carry particularly strong risk for violence/homicide? Third, does downward **social mobility** (becoming poor) increase risk for violence/homicide? Table 1 explains the hypotheses related to these research questions.

**Table 1.** Description of life-course hypotheses about the influence of poverty on violence

Life course hypothesis	Description	Test
Accumulation	An accumulation hypothesis states that the cumulative sum of exposure (the number of times an individual is exposed to poverty) is the best explanation for the outcome.	Strength of association between the number of times the cohort participant is exposed to poverty (summing across birth, ages 4 and 22 years) and violence.
Critical period	A critical period hypothesis states that the exposure is only associated with the outcome during one period of exposure.	Comparison of the poverty-violence association considering each different age of exposure to poverty, to identify any unique age at which poverty is associated with violence (considering poverty exposure at birth, age 4 years and age 22 years).
Sensitive period	A sensitive period hypothesis states the exposure-outcome association is stronger in a particular period of exposure. For example, even if there are associations between poverty and violence at other ages, the strongest association might be observed for poverty exposure at the time of birth, in which case birth would represent a sensitive period.	Examination of whether exposure to poverty at any single age (out of birth, age 4 and age 22) has a particularly strong association with violence, when other ages may have weaker associations or an accumulation effect is also observed.
	Note that the sensitive period hypothesis is a ‘compound’ hypothesis involving multiple exposures associating with the outcome (such as accumulation of poverty and effects at a specific age). The defining feature is that poverty at one age has a stronger association with violence than at other ages.	
Downward mobility	A mobility hypothesis states that the outcome is associated with changes in the exposure over time. We examine the effect of becoming poor between birth/early childhood and young adulthood.	Strength of association between violence and a change in poverty from not poor to poor (tested in relation to change from ages birth to 22 years and from 4 years to 22 years).

## Methods

The 1982 Pelotas Birth Cohort Study is one of the largest and longest running prospective cohorts in the Global South. Pelotas is a relatively poor, Southern Brazilian city with an estimated population of 340 000 inhabitants. All live-born children delivered in 1982 in the city’s hospitals (99% of all births took place in hospitals), and their mothers, were included in the study (n = 5914). We used data on household income from assessments at birth, in early childhood (age 4 years; 84.1% retention), and in early adulthood (age 22 years; 78.9% retention), and examined crime records searched for all cohort members at age 30 years. Further study information is detailed elsewhere.^22^^,^^23^

**Figure 1.**
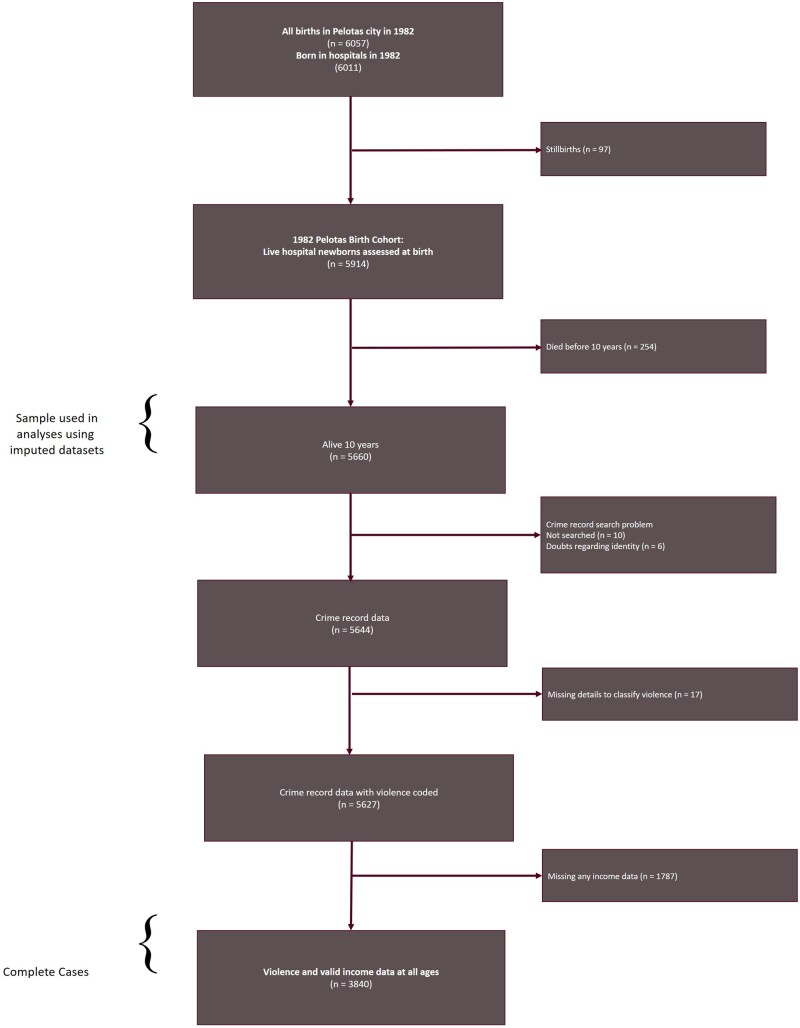
Flow diagram of the 1982 Pelotas Birth Cohort Study and linked crime records

Of 5660 participants still alive at age 10 years (eligible for analyses), 5627 (99.4%) had valid crime data on violence (there were 16 unsuccessful record searches/identity doubts, and 17 cases had insufficient details for classifying violence). Of those, 3840 had complete life-course income data (complete case sample). We also conducted imputed analyses for all 5660 participants alive at age 10 years (see Figure 1).

### Measures of violence and homicide

Official crime records were obtained when cohort members were median age 30.7 years. Details about the searches are in the Supplementary Material (available as Supplementary data at *IJE* online). Two binary outcome variables were derived: any violent offending (vs no violent offending), and any homicide (vs no homicide). These refer to records of suspected offences at any age.

In sensitivity analyses, we examined additional outcomes. For clear temporal precedence, we examined offences committed after age 22 years (main analyses had no restriction on age). To consider potential bias regarding use of official records, we also examined self-reported data on fights in the past year, coded as a binary variable (self-reported fight at either 22 or 30 years vs none at both time points).

### Measures of poverty

Household income data were collected at birth, and at ages 4 and 22 years. The parameter commonly used in Brazil to measure poverty is in terms of Brazilian monthly minimum wages [BMMW^24^]. We followed previous procedures^25^ for these income data, defining poverty as having household income <3 BMMW, considering a threshold suggested by the Brazilian government^24^ and evidence this identifies children at particular risk for not achieving their developmental potential.^26^^,^^27^ In sensitivity analyses, we also examined poverty defined as <1 BMMW, and relative poverty defined as the lowest income tertile at each age (see Supplementary Material, available as Supplementary data at *IJE* online for further details).

### Measures of covariates

Potential confounders for the association between poverty and violence/homicide were: sex, maternal education (≤4 years completed schooling vs > 5), and maternal age (<20 years vs higher), all measured in perinatal interviews with the mother. For the association between violence/homicide and poverty in young adulthood, maternal education and age represent potential confounding factors. For poverty in early childhood, they could represent either potential confounding or mediating mechanisms.

### Statistical analysis

Following a prespecified analysis plan (see https://osf.io/bvs7z/), we examined the influence of poverty on violence/homicide in the following steps. First, we characterised the total birth cohort (*n* = 5914), participants alive at age 10 years (*n* = 5660), and the complete case sample (*n* = 3840), using sociodemographic data collected at birth. Second, for those with valid crime data (*n* = 5627), we describe the violence and homicide records in the cohort. Third, we present rates of poverty at each age, and continuity of poverty through time, before examining associations with violence/homicide, calculating odds ratios (OR) and 95% confidence intervals (95% CI), using logistic regression. We conducted both complete case analyses (*n* = 3840) and analyses based on multiple imputation producing a sample size of 5660, representing participants alive at age 10 (Figure 1). Imputation models included all study variables (details in Supplementary Material, available as Supplementary data at *IJE* online). Results based on imputed data were prioritised due to some small cell counts for complete cases.

### Testing life-course models

To test life-course hypotheses, we used a structured life course modelling approach (SLCMA) with logistic regression.^28–30^ SLCMA identifies the best-fitting model based on the least absolute shrinkage and selection operator.^30^ Advantages of an SLCMA relative to other methods (e.g. stepwise regression; structural equation modelling) include that it identifies the most parsimonious model for the outcome, while comparing competing theoretical life-course models simultaneously. The approach also provides statistical inference not biased by selecting the most parsimonious model.

A set of key variables encoding each theoretical life-course hypothesis (see Table 1) were entered into crude least absolute shrinkage and selection operator models, and models adjusted for sex, maternal education and maternal age. We used elbow plots to determine the number of key variables to include and identify the most plausible (best-fitting) life-course model,^31^ repeating the process for any violence and homicide separately.

To estimate effect sizes (and confidence intervals and *P-*values) in final models, we used bootstrapping on the stacked imputed datasets,^32^ and selective inference,^33^ supplemented by Bonferroni-corrected inference, for the complete case analyses, which control for family-wise error rates.^33^^,^^34^ All analyses were conducted in R (version 4.1.0) and reproducible code will be made open access via the Open Science Framework.

### Deviations from the statistical analysis plan

Two analyses in the pre-specified analysis plan were not completed for reasons detailed in the Supplementary Material (available as Supplementary data at *IJE* online), and one additional sensitivity test (regarding relative poverty) was added.

## Results

Just over two-thirds of families were living in poverty at the start of the study, in 1982. Cohort participants alive at age 10 years (*n* = 5660; analysed in imputed datasets) and participants with complete poverty and crime data (*n* = 3840) each had very similar baseline sociodemographic characteristics and rates of violence, compared with the whole cohort (Table 2).

**Table 2.** Sample characteristics of the 1982 Pelotas Birth Cohort at baseline, compared between total cohort, imputed sample and sample with complete poverty and violence data

	Total cohort	**Imputed sample** ^a^	**Complete case sample** ^b^
	(*n* = 5914)	(*n* = 5660)	(*n *= 3840)
Sex			
Female	2876 (48.6%)	2762 (48.8%)	1861 (48.5%)
Male	3037 (51.4%)	2898 (51.2%)	1979 (51.5%)
Missing	1	0	0
Maternal age			
<20	911 (15.4%)	855 (15.1%)	540 (14.1%)
20+	5002 (84.6%)	4804 (84.9%)	3300 (85.9%)
Missing	1	1	0
Maternal education			
≤4 schooling years	1960 (33.2%)	1835 (32.5%)	1248 (32.5%)
5 + schooling years	3947 (66.8%)	3817 (67.5%)	2590 (67.5%)
Missing	7	7	2
Family income at birth			
Poor	4077 (69.3%)	3860 (68.5%)	2673 (69.6%)
Not poor	1808 (30.7%)	1774 (31.5%)	1167 (30.4%)
Missing	29	26	0
Violent crime			
Yes	1151 (20.5%)	1151 (20.5%)	839 (21.9%)
No	4476 (79.6%)	4476 (79.6%)	3001 (78.2%)
Died by age 10	254	0	0
Missing	33	33	0

Column percentages are based on cases with valid data.

a Individuals still alive at 10 years.

b Individuals still alive at 10 years, with valid violence and poverty data.

Of 5627 cohort members alive at age 10 years with valid data on violence (Figure 1), 20.5% (1151) had a record for at least one violent crime when records were searched (median age 30.7 years). Of those, 48 were suspected homicide offenders (*n* = 45, 93.8% male), and 1103 were suspected of non-lethal violent crimes only (*n* = 750, 68.0% male).

**Figure 2.**
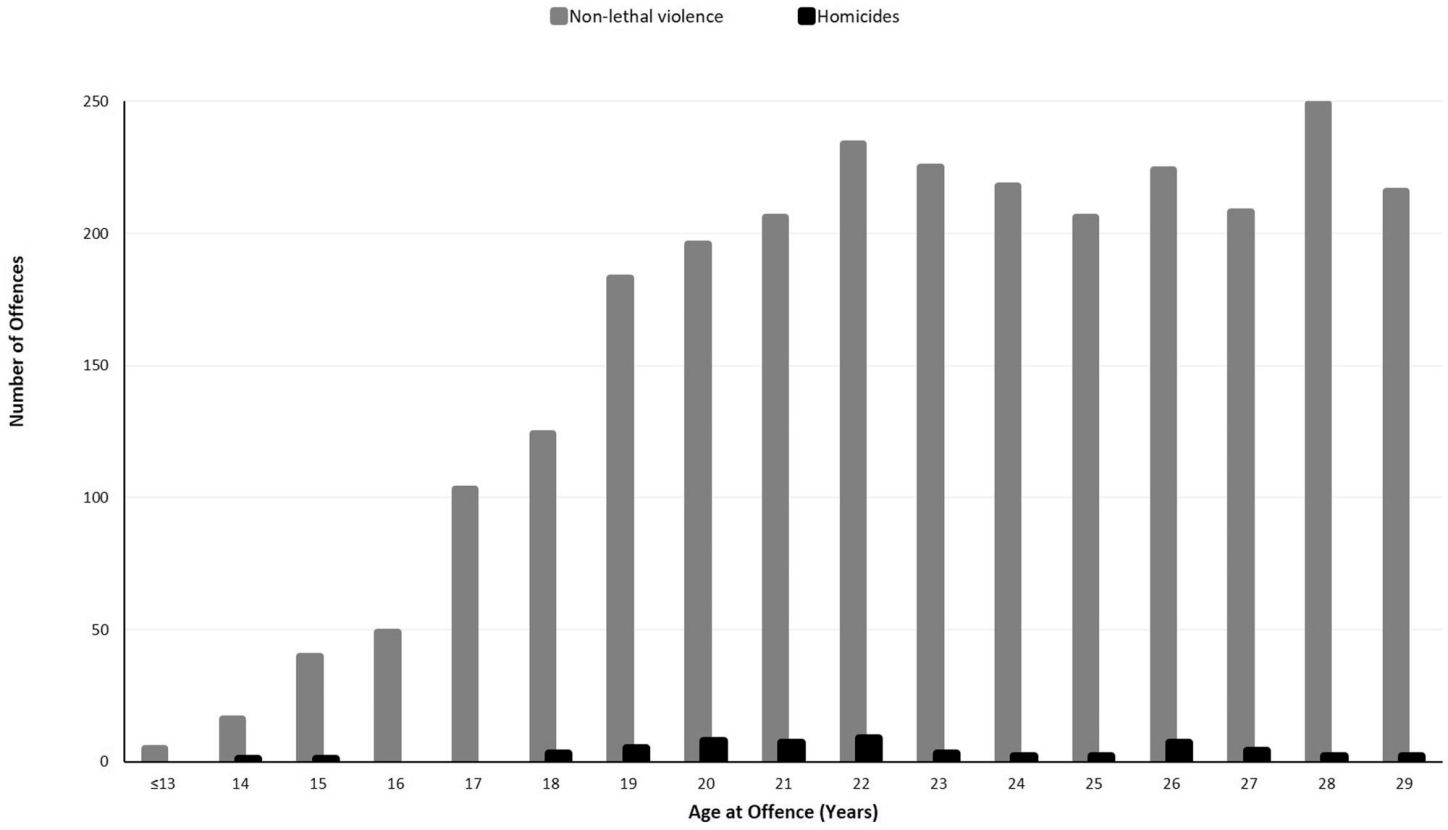
Age-crime curve for homicide and non-lethal violence in the 1982 Pelotas Birth Cohort (*n* = based on 5627 participants with valid violence data; 2907 offences registered by 1151 people). Note, figure restricted to years with complete 12 months of data—up to age 30.with valid violence data

In total, 2907 violent offences were recorded in the cohort, including 58 homicides and 2849 non-lethal violent offences. Figure 2 shows the distribution of offences by age, known as the ‘age-crime curve’, with a notable persistence in offending until the end of the study period. Further details about the average number of crimes per offender and average age of offences are in the Supplementary Table S1 (available as Supplementary data at *IJE* online).

Table 3 shows characteristics of the 58 homicide offences—30.4% were completed homicides and the remainder attempted (we refer to both as homicide offences in this article). Most homicides (62.2%) were committed using a firearm, and the majority (55.6%) occurred on the street. Few (5.2%) homicide victims were identified as partners of the offender, although for 34.5% of cases, the relationship between victim and offender was unknown to the police. Many homicide offences did not reach the courts (36.2%), and few cases led to convictions (15.5%).

**Table 3.** Characteristics of homicide offences (*n* = 58) in the 1982 Pelotas Birth Cohort

	Total
Offender sex	
Male	55 (94.8%)
Female	3 (5.2%)
Brazilian legal classification	
Homicide	56 (96.6%)
Latrocínio	2 (3.4%)
Completed/attempted homicide	
Attempted	39 (69.6%)
Completed	17 (30.4%)
Age at offence	
10–17	2 (3.5%)
18–23	35 (60.3%)
24–30	21 (36.2%)
Use of weapon	
Firearm	23 (62.2%)
Sharp object	7 (18.9%)
No weapon	7 (18.9%)
Relationship to victim	
Spouse/partner/boyfriend	3 (5.2%)
Other person known to offender	31 (53.5%)
Not known by offender	4 (6.9%)
Not identified by police	20 (34.5%)
Context	
Domestic	9 (25.0%)
Street	20 (55.6%)
Other^a^	7 (19.4%)
Criminal outcome	
No court record	21 (36.2%)
Convicted	9 (15.5%)
Archived/prescribed	12 (20.7%)
Acquitted	16 (27.6%)

Column percentages. Note data could not be coded for lethal offences in relation to attempted/completed homicide (*n *= 2), use of weapon (*n* = 21), context (*n *= 22). Latrocínio is a separate lethal offence category in Brazil, meaning homicide followed by theft of property, but both homicide and latrocínio are referred to as homicides in this article.

a Other includes business location, institution or transport.

### Poverty and violence: simple, bivariate associations

Exposure to poverty reduced from time of birth (69.6%) to early childhood (58.5% age 4 years) and young adulthood (37.0% age 22 years). However, there was significant continuity in poverty: among 2673 children poor at birth, 58.4% were also poor in early childhood (OR = 13.15; 95% CI: 11.29, 15.32), and 37.0% were poor in young adulthood (OR = 3.99; 95% CI: 3.40, 4.68). ‘Chronic poverty’—at birth, early childhood and early adulthood–was experienced by 26.1%. More details about continuity in poverty through time are in the Supplementary Material (Tables S2 and S3, available as Supplementary data at *IJE* online).

**Figure 3.**
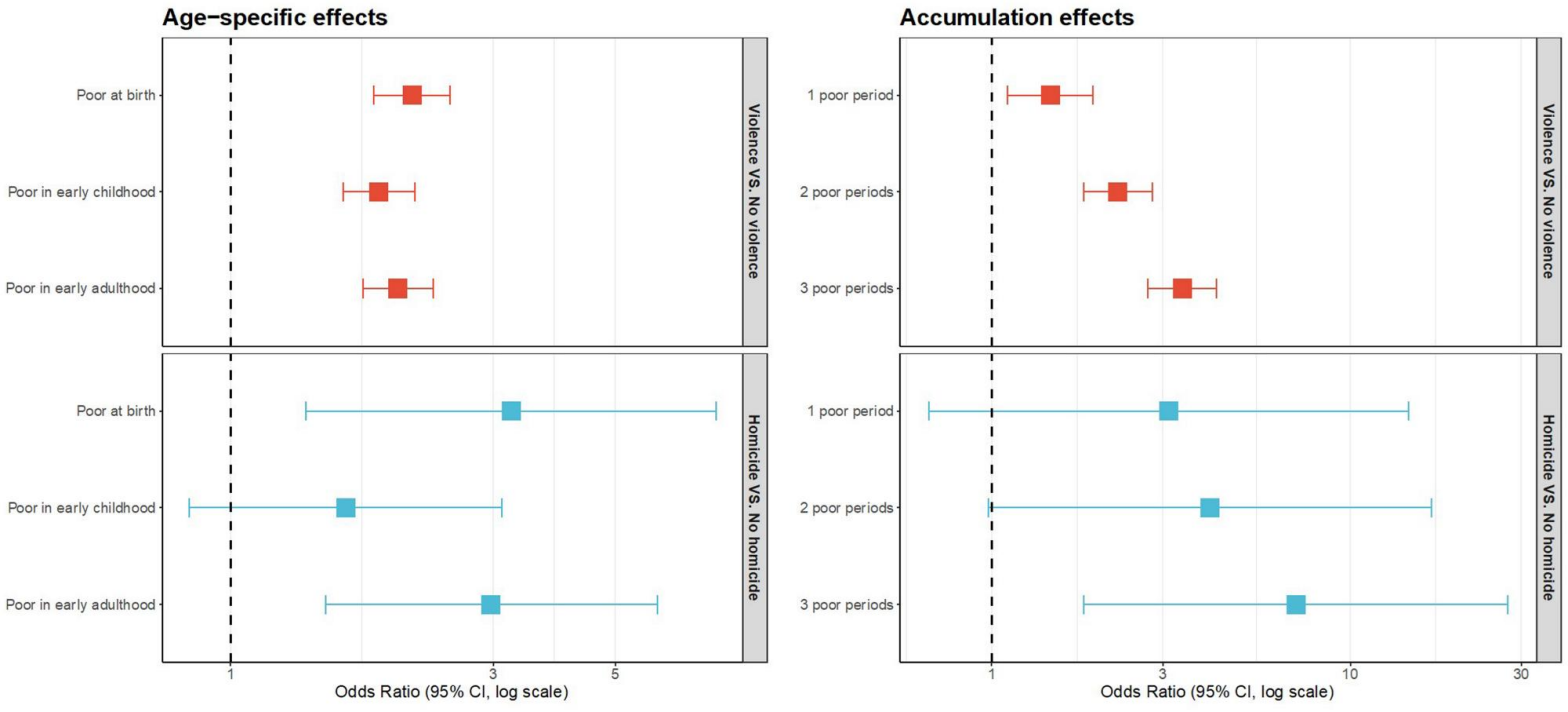
Bivariate associations of poverty with any violence, and homicide specifically (imputed analyses, *n *= 5660)

Table 4 shows elevated rates of both homicide and non-lethal violence among participants exposed to poverty at each age (based on complete data; *n* = 3840). Figure 3 shows bivariate associations between poverty at each age and violence and homicide, using multiple imputation to handle missing data. Exposure to poverty at birth, in early childhood and in early adulthood approximately doubled the odds of any violence, and the association with homicide was large (e.g. OR = 3.24; 95% CI: 1.37, 7.63, for poverty at birth; OR = 2.97, 95% CI = 1.49, 5.96, for poverty in early adulthood) (see Supplementary Table S4, available as Supplementary data at *IJE* online for further numeric results).

**Table 4.** Proportion of 1982 Pelotas Birth Cohort with violent outcomes according to poverty status at birth, early childhood and early adulthood (complete cases, *n* = 3840)

		No violence	Non-lethal violence	Homicide
	*N*	*n* (%)	*n* (%)	*n* (%)
Poverty at birth				
Not poor	1167	1004 (86.0%)	159 (13.6%)	4 (0.3%)
Poor	2673	1997 (74.7%)	650 (24.3%)	26 (1.0%)
Poverty early childhood				
Not poor	1592	1341 (84.2%)	242 (15.2%)	9 (0.6%)
Poor	2248	1660 (73.8%)	567 (25.2%)	21 (0.9%)
Poverty early adulthood				
Not poor	2420	2001 (82.7%)	407 (16.8%)	12 (0.5%)
Poor	1420	1000 (70.4%)	402 (28.3%)	18 (1.3%)
Number of ages poor^a^				
0	829	730 (88.1%)	97 (11.7%)	2 (0.2%)
1	684	569 (83.2%)	110 (16.1%)	4 (0.6%)
2	1324	1016 (76.7%)	297 (22.4%)	11 (0.8%)
3	1003	685 (68.3%)	305 (30.4%)	13 (1.3%)

Row percentages.

a Count of poverty exposure across birth, early childhood and early adulthood.

### Life-course models of poverty and violence

**Figure 4.**
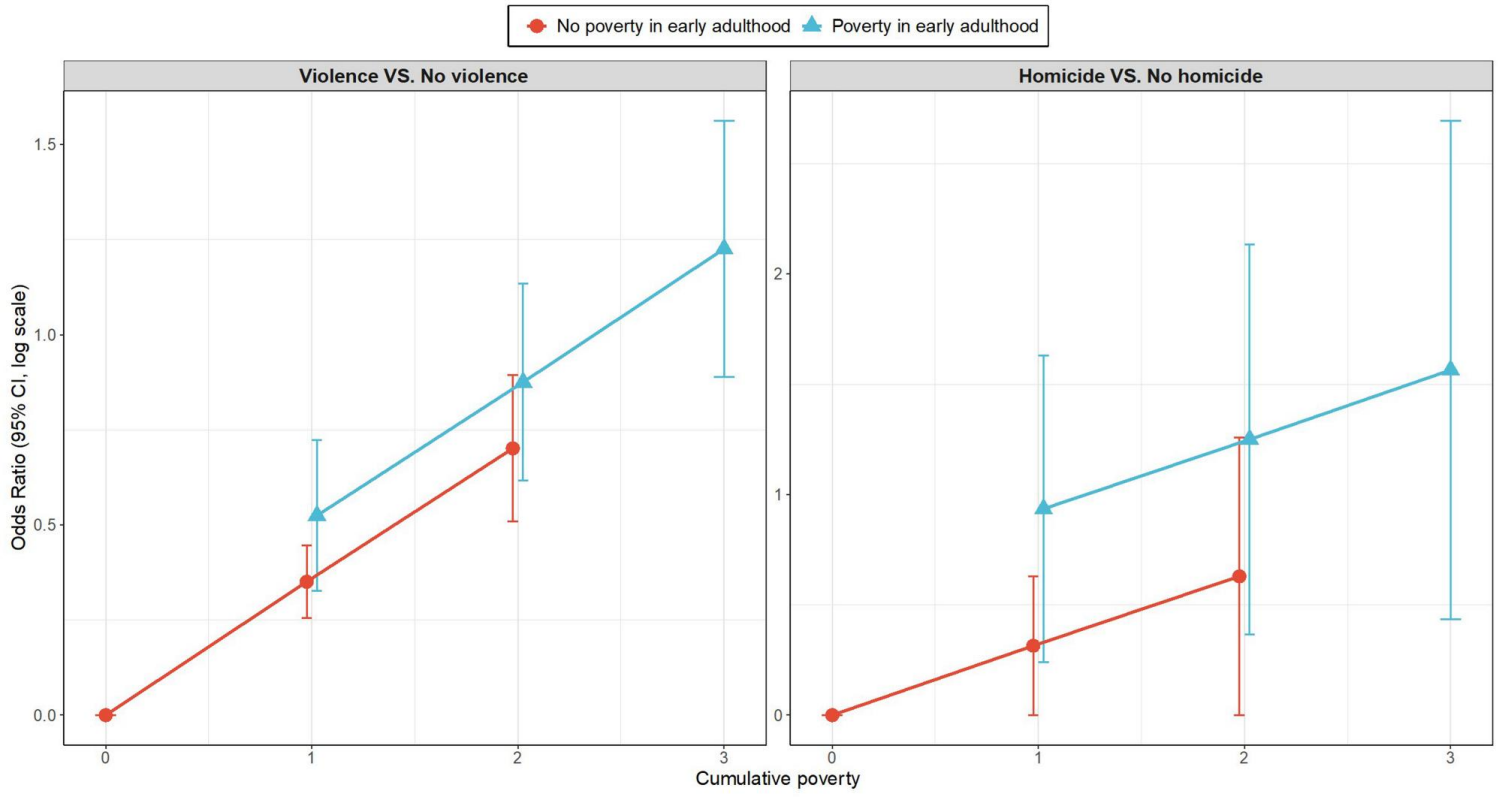
Effects of both cumulative poverty and poverty in early adulthood on violence/homicide, showing that early adulthood is a sensitive period for the effects of poverty. Estimates based on imputed, unadjusted analyses (*n* = 5660)

There was strong evidence in SCLMA modelling that all violence was best explained by a combination of both cumulative poverty and poverty in early adulthood, implying that early adulthood is a sensitive period for the effects of poverty on all violence. Adjusting for sex, maternal age and maternal education at birth, cumulative poverty increased risk for violence by 33% (OR = 1.33; 95% CI: 1.20, 1.46), and poverty in early adulthood additionally increased risk by 35% (OR = 1.35; 95% CI: 1.11, 1.64) (results based on multiple imputation, Table 5). Unadjusted models using imputed data also supported the hypothesis of a sensitive period in early adulthood. Figure 4 shows the relative importance of poverty in adulthood and cumulative poverty, in unadjusted analyses of imputed data.

**Table 5:** Life-course models of the influence of poverty on all violence and homicide in the 1982 Pelotas Birth Cohort (imputed sample, *n* = 5660)

		Adjusted		Unadjusted	
		Type of poverty exposure	Association with outcome	Type of poverty exposure	Association with outcome
			OR (95% CI)		OR (95% CI)
Violence					
First variable		Accumulation	1.33 (1.20, 1.46)**	Accumulation	1.42 (1.29, 1.53)**
Second variable		Poverty early adulthood	1.35 (1.11, 1.64)*	Poverty early adulthood	1.19 (1.00, 1.44)
Homicide					
First variable		Poverty early adulthood	2.85 (1.00, 4.89)	Poverty early adulthood	1.86 (1.00, 4.03)
Second variable		Poverty at birth	2.51 (1.00, 8.30)	Accumulation	1.37 (1.00, 1.94)

Odds ratios (OR) and 95% confidence intervals (CIs) estimated using bootstrapping on 20 multiply imputed datasets by chained equations (MICE). *P* values derived via bootstrapped confidence intervals (95% and 99%). Adjusted models adjust for sex, maternal age and maternal education, which were all measured at birth.

* *P* <0.05,

** *P* <0.01.

For homicide specifically, there was also evidence that early adulthood was a sensitive period. In fact, for homicide (unlike all violence) the association with poverty in early adulthood was selected first as the most important predictor in SCLMA modelling (OR = 2.85; 95% CI = 1.00, 4.89, in adjusted models, Table 5), followed by accumulation in the unadjusted model, and by poverty at birth in the adjusted model.

Analyses of complete cases also supported the hypothesis of a sensitive period in young adulthood for both violence and homicide specifically (Supplementary Table S5, available as Supplementary data at *IJE* online). Sensitivity analyses showed the main results were robust to various specifications of poverty and violence (see Supplementary Material, available as Supplementary data at *IJE* online).

## Discussion

In this prospective, population-based birth cohort study of over 5000 people in Brazil, poverty in early adulthood was strongly associated with all criminal violence as well as with homicide specifically. Poverty in early life (at birth and in early childhood) was also associated with violence, as was cumulative poverty. However, early adulthood was the most important period for the effects of poverty on violence and homicide, yielding support for a sensitive period hypothesis at that age. Notably, 1.6% of males in this study were suspected homicide offenders by age 30 years. To our knowledge, this is the only prospective study outside the USA to examine the association between poverty and homicide, and the only study worldwide to test competing life-course hypotheses about poverty and violence.

Although a longitudinal link between poverty and non-lethal violence has been demonstrated in high-income countries,^6^^,^^35^ evidence is scarce in the Global South, where the highest rates of serious violence are found.^36^ As such, the current study fills an important gap in knowledge, showing that poverty in Brazil is linked to violence of varying levels of severity, from self-reported fights to criminal violence in official records, as well as homicide.

Potential mechanisms between poverty and violence, and prevention issues, are considered here, recognizing that implications are speculative. Key theories emphasize that lack of legal opportunities to work and generate income are important to understanding criminal violence,^16^^,^^17^ implicating mechanisms of frustration and anger, as well as material needs. The context of youth poverty in Brazil suggests additional factors involved. In particular, poverty often occurs in segregated neighbourhoods with low state support, ineffective and violent policing, discrimination, and control by gangs involved in drug trafficking.^37^^,^^38^ In other contexts – in most previous studies in other countries – a natural decline in crime is expected during the transition to adulthood, after peaking in late adolescence, as shown in the classic ‘age-crime curve’.^39^ By contrast in the current study, frequent violent offending was observed until age 30 years (Figure 2). We suggest this unusual pattern arose because of the specific social context in Brazil^37^^,^^38^–prolonging adverse effects of poverty on violence through young adulthood.

Youth education, employment and welfare programmes are obvious policy responses to reduce youth poverty and associated violence. Such support is particularly needed for those already involved in the criminal justice system, as well as at-risk youth, for example those failing in school.^40^ Although evidence-based crime prevention is limited in Brazil, the Pelotas government is implementing projects to support educational achievement and employment opportunities for at-risk youth and ex-prisoners, embedded in a wider intersectoral violence prevention plan, with promising first results.^41^ However, structural determinants of poverty and violence also demand wider policy reforms at state and federal levels.

Only 15.5% of homicide cases in the current study led to a criminal conviction, and a third did not even reach the courts, reflecting widespread immunity from punishment in Brazil,^42^ which is also considered a key determinant of crime. Two-thirds of homicides in this study were committed with a firearm, similar to national rates^43^; evidence suggests that limiting the availability of firearms could considerably reduce rates of homicide.^36^^,^^44^^,^^45^

Although early adulthood was the most important life period for the link between poverty and violence in this study, cumulative poverty, including exposure in early life, was also influential. Support to vulnerable families with young children^46^ is important to mitigate intergenerational transmission of social disadvantage, and potentially prevent violence. Conditional cash transfer programmes like ‘Bolsa Família’ and large-scale home visiting programmes for child development^47^^,^^48^ are notable policies towards this end in Brazil, and are recommended by the World Health Organization for reducing violence.^49^

The current study brought a powerful, new analytical approach to examine the association between poverty and violence (SCLMA). Despite a wealth of longitudinal research on violence, studies have mainly conducted cross-sectional analyses of risk factors, and we are not aware of any prior study that used SLCMA. The current study is also one of the first successful uses of SLCMA with binary outcome variables, suggesting its utility for a wide range of applications in life sciences.

The study is not without limitations. Despite it being a large cohort in a high-violence context, the number of homicide offenders (*n* = 48) limited statistical power. The main outcome variables (violence and homicide) were measured using criminal records indicating suspected offenders, involving some misclassification. Bias towards recording disadvantaged people as crime suspects would inflate estimated associations, although this limitation is somewhat mitigated by similar results obtained for self-reported fights. Although a relatively high follow-up rate was attained, there were significant missing self-report data in young adulthood. This could have biased the results, although this does not seem likely given that the analytical sample was very similar to the whole cohort in both baseline characteristics and later violence. The study did not include measures of poverty during adolescence, which might be another sensitive period regarding effects on violence. The data are observational, not experimental, limiting causal inference. Finally, the study was conducted in a single municipality in Southern Brazil, and it cannot be assumed that findings generalise across the country.

## Conclusion

In conclusion, exposure to poverty is strongly associated with criminal violence and homicide in this Brazilian population, with the strongest influence identified for poverty in early adulthood. Policies to reduce poverty and violence, and tackle their adverse social contexts, are urgently required in Brazil.

## Ethics approval

The Research Ethics Committee of the Faculty of Medicine, Federal University of Pelotas, approved all assessments of the 1982 Pelotas Birth Cohort (latest approval, age 30: #16/12). Written informed consent was obtained from participants before interview. For the crime record linkage project, ethical approval was obtained from the Research Ethics Committee of the Faculty of Medicine, Federal University of Pelotas (#08/12) and the Institute of Criminology, University of Cambridge (21/5/2009).

## Supplementary Material

dyae103_Supplementary_Data

## Data Availability

This article is based on data from the 1982 Pelotas Birth Cohort Study conducted by the Postgraduate Program in Epidemiology at the Federal University of Pelotas with the collaboration of the Brazilian Public Health Association (ABRASCO). The questionnaires and interviewer guides from all follow-up visits are available in electronic formats at [https://epidemioufpel.com.br/index.php/coorte-1982/]. The cohort profile^22^ explains procedures for accessing data from the study via contact with the study coordinators and completion of a data access request form available at [http://www.epidemio-ufpel.org.br/site/content/downloads/index.php].
