# Supplementary material for: Life-course influences of poverty on violence and homicide: 30-year Brazilian birth cohort study
Source: Int J Epidemiol. 2024 Aug 9;53(4):dyae103. doi: 10.1093/ije/dyae103 (PMC11315650; doi:10.1093/ije/dyae103)
Supplement: dyae103_Supplementary_Data [file dyae103_supplementary_data.pdf]

Life-course influences of poverty on violence and homicide: 30-year Brazilian birth cohort study

Joseph Murray, Michelle Degli Esposti, Christian Loret de Mola, Rafaela Martins, Andrew D.A.C. Smith, Terrie E. Moffitt, Jon Heron, Vanessa Iribarrem Miranda, Natalia Lima, Bernardo L. Horta

SUPPLEMENTARY MATERIAL

Details of Crime Records

For linked crime records, all criminal records of suspected offences for cohort members were searched up until March 2013, when participants were median age 30.7 years (range: 30.2-31.2). We searched police records in Rio Grande do Sul's state integrated security system (*Secretaria de Segurança Pública*, SSP), court records in the state's judiciary system (*Poder Judiciário*), records from the young offender institutions (*Centro de Atendimento Sócio-Educativo* - CASE - and *Fundação de Atendimento Sócio-Educativo* - FASE) and adult prisons, identifying any record relating to a participant as suspect of a crime, either as a minor or adult.

Record linkage was based on the cohort participant's name, parents' names, and date and place of birth, and apart from the SSP (where automated searches were possible) was done manually by searching for each individual cohort participant in the relevant system. In the SSP, all cohort participants were identified in the state integrated security system, as either having no criminal record, or details of each police record pertaining to the individual. In the other criminal record systems, individual details were entered manually by a member of the research team, who either recorded that no records were located for that individual, or all pertaining records were extracted for subsequent coding.

As described in the Methods of the main article, of the 5660 cohort participants alive at age 10 years (eligible for this study), successful linkage with crime records was achieved for 5644 participants (99.7%), with 16 unsuccessful record searches or identity doubts. Of these 5627 (99.4% of 5660) had sufficient details in crime records to code whether a violent offence had been committed or not.

Crime records that met the definition of a crime or criminal contravention in Brazil were retained for study, except for abortion. Crimes are considered more serious illegal acts, and evoke a punishment of imprisonment up to 30 years and/or a fine, according to Brazilian law, both for completed and attempted illegal acts. Criminal contraventions are less serious law violations and evoke a simple prison sentence (in open or semi open prisons) up to five years and/or a fine, and only in the case of completed (not attempted) illegal acts.

Cleaning and coding of crime records was completed over a nine-year period from 2013. For each offence, information on the involvement of the cohort member, crime type, and date of crime was manually extracted and coded by two trained law students using all available data (from police, courts, young offender institutions, and prisons), blind to each other's classifications. The cohort member was initially identified as the victim, witness, or offender for each offence, and the crime type was then classified by Brazilian law. For the current study, only records of suspected offenders (not victims or witnesses) were examined. Legal criminal categories were later condensed to create broader crime categories for analyses, including homicide, non-lethal violence, and non-violent crime. The homicide outcome includes all completed and attempted lethal violence, classified in Brazilian law either "homicide" or "*latrocínio*" — a separate lethal offence category meaning homicide followed by theft of property. Both attempted and completed homicides and "*latrocínio*" offences, are referred to as homicide offences in this article.

The date of crime was taken directly from the records, and where those were occasionally missing, other dates (e.g., date of police investigation or court record) were used to estimate the date of offence, based on average periods between offences and these criminal justice procedures. Since there were often multiple sources of information on the same crime (e.g., from the police and courts), information from the courts were prioritised as these were believed to be a more reliable source. Any classification discrepancies were discussed on a case-by-case basis and resolved by consensus or via the inclusion of a third lawyer.

Details of income data

Monthly income data in the 1982 cohort were collected in interviews and referred to the total amount of household earnings in the month before the interview. The parameter commonly used in Brazil to measure poverty is in terms of Brazilian monthly minimum wages [BMMW; 1]. These data were collected with mothers (birth, age 4) and with cohort members (age 22) in continuous level form (expressed in local currency and converted into multiples of the BMMW at the time of the of data collection), except for at birth, when data were originally collected according to five categories of BMMW multiples (≤1.0, >1.0 to 3.0, >3.0 to 6.0, >6.0–10.0 and >10).

Further details on statistical analyses

Multiple imputation

Imputation models included all study variables. We used multiple imputation by chained equations (MICE) to impute missing data for maternal age (0.02%), maternal education (0.12%), family income at birth (0.46%), family income at 4 years (16.63%), family income at 22 years (24.13%), and violent crime (0.58%), producing 20 datasets with a sample size of 5660, representing all cohort members alive at age 10 years. In line with previous recommendations [2, 3], analyses were carried out across the 20 imputed datasets and graphical and internal checks indicated that these imputed data were reasonable. Therefore, additional imputations were not carried out since these would have incurred substantial computational costs (given the subsequent use of bootstrapping on the stacked imputed datasets).

Deviations from the pre-specified analysis plan

The pre-specified analysis plan was carefully followed, with three adjustments. First, we had planned to create a third, additional outcome variable coded 1 for homicide offender, and 0 for non-lethal violent offender. However, recognising that analysing this outcome variable (excluding participants without violent offences) could result in poor estimates because of collider bias, this third outcome variable was not analysed. Second, we did not conduct planned sensitivity analyses restricting the outcome to cases of conviction, because of the low number of offences that resulted in conviction. Third, considering the changing levels of absolute poverty (defined as < BMMW) between birth and early adulthood, we ran an additional sensitivity analysis of relative poverty (defined as bottom third in income distribution at each age).

Findings from sensitivity analyses

Sensitivity analyses identified that the main results were robust to various specifications. Defining poverty as <1 BMMW (compared to <3 BMMW in the main models) also identified early adulthood as a sensitive period for the association between poverty with all violence (Supplementary Table S6; note, homicide analyses could not be conducted robustly given small cell counts for this definition of poverty). Defining poverty in relative terms, as being in the bottom income tertile at each age, also identified early adulthood as a sensitive period for the association between poverty with both all violence and homicide (Supplementary Table S7).

Additionally, restricting official records to violent offending after 22 years old to establish complete temporal precedence (early adulthood poverty occurring before all offending) further supported early adulthood as a sensitive period, yielding stronger evidence of possible causality (Supplementary Table S8).

A sensitivity analysis using self-reported violence (fights) as the outcome also isolated early adulthood as an important exposure period for the influence of poverty (Supplementary Table S8). However, unlike the main models which used official records, there was limited support for cumulative effects of poverty on self-reported violence. Instead, additional specific effects of downward mobility were indicated, where individuals who experienced a shift from no poverty in early childhood to poverty in early adulthood were at increased risk of self-reporting violent outcomes.

Average number of offences and age of offences

Supplementary Table S1. Average number of offences and average age of offences among violent/homicide offenders in the 1982 Pelotas Birth Cohort (n=1151)

	**Total**				**Males**				**Females**			
	**Total number offences***		**Age first offence****		**Total number offences***		**Age first offence****		**Total number offences***		**Age first offence****	
**Type Offender**	**Mean (sd)**	**Median [range]**	**Mean (sd)**	**Median [range]**	**Mean (sd)**	**Median [range]**	**Mean (sd)**	**Median [range]**	**Mean (sd)**	**Median [range]**	**Mean (sd)**	**Median [range]**
**Non-lethal violent** (n=1103)	3.1 (0.12)	2.0 [1-24]	21.9 (0.14)	21.0 [9.0-30]	3.6 (0.16)	2.0 [1-24]	21.4 (0.18)	21.0 [9.0-30]	2.0 (0.0)	2.0 [2-26]	22.6 (0.23)	22.0 [13.0-30]
**Homicide** (n=48)	1.42 (0.09)	1 [1-4]	22.9 (4.0)	23.0 [13.0-31.0]	1.38 (0.10)	1 [1-4]	22.9 (3.9)	23.0 [13.0-31.0]	1.67 (0.33)	2 [1-2]	23.6 (4.4)	23.0 [14.0-30.0]

Note: Standard deviation (sd)
*Total number offences refers to the specific type of offence for that offender (e.g. the total number of homicide offences committed by each homicide offender; where mean = 1.42).
**Age at first offence also refers to the age at which the specific type of offence (of the offender) was first committed (e.g. the age at first homicide offence among homicide offenders, where median = 22.9).

Poverty exposure and continuity in poverty

Supplementary Table S2 Prevalence of poverty at each period of the life-course, and continuity in poverty through time (n=3840)

		Association with poverty early childhood	Association with poverty early adulthood
	**n poor (%)**	**OR (95% CI)**	**OR (95% CI)**
**Poor at birth**	2673 (69.6%)	13.15 [11.29, 15.32]***	3.99 [3.40, 4.68]***
**Poor in early childhood**	2248 (58.5%)	..	3.49 [3.02, 4.03]***
**Poor in early adulthood**	1420 (37.0%)	..	..

Note: Odds ratio (OR); 95% CI (95% confidence interval)
Row percentages. ****P* <0.001

Supplementary Table S3 Cumulative number of ages poor in the 1982 Pelotas Birth Cohort (n=3840)

Number of ages poor†		Poor at birth	Poor early childhood	Poor early adulthood
	**N (%)**	**n (%)**	**n (%)**	**n (%)**
**0**	829 (21.6%)	0 (0%)	0 (0%)	0 (0%)
**1**	684 (17.8%)	428 (62.6%)	137 (20.0%)	119 (17.4%)
**2**	1324 (34.5%)	1242 (93.8%)	1108 (83.7%)	298 (22.5%)
**3**	1003 (26.1%)	1003 (100%)	1003 (100%)	1003 (100%)

Row percentages. †Count of poverty exposure across birth, early childhood, and early adulthood.

Poverty exposure and violence outcomes

Supplementary Table S4. Bivariate associations between poverty through the life-course and violence (imputed analyses, n=5660)

		**Violence versus No violence**	**Homicide versus No homicide**
		**OR (95% CI)**	**OR (95% CI)**
**Poor at birth**		2.14 [1.82, 2.5]***	3.24 [1.37, 7.63]**
**Poor in early childhood**		1.86 [1.6, 2.16]***	1.62 [0.84, 3.11]
**Poor in early adulthood**		2.01 [1.74, 2.33]***	2.97 [1.49, 5.96]**
**Number of ages poor†**			
	0	Ref.	Ref.
	1	1.46 [1.11, 1.92]**	3.12 [0.67, 14.55]
	2	2.25 [1.81, 2.81]***	4.06 [0.98, 16.83]
	3	3.40 [2.73, 4.24]***	7.05 [1.81, 27.49]**

Note: Odds ratio (OR); 95% CI (95% confidence interval)
Results based on multiple imputation by chained equations (20 imputed datasets) with a sample size of 5660 of those who were still alive at 10 years.
†Count of poverty exposure across birth, early childhood, and early adulthood
**P* <0.05; ***P* <0.01; ****P* <0.001

Supplementary Table S5. Unadjusted model selection and effect estimates from fixed Least Absolute Shrinkage and Selection Operator inference (selective inference and Bonferroni-corrected logistic regression) using complete cases (n = 3840)

	**Type of poverty exposure**	**Selective inference Association with outcome**		**Conservative logistic regression Association with outcome offending**	
		**OR (95% CI)**	***P* value**	**OR (95% CI)**	***P* value**
**Violence (versus no violence)**					
**First variable**	Accumulation	1.44 (1.29, 1.65)	<0.001	1.44 (1.29, 1.60)	<0.001
**Second variable**	Poverty early adulthood	1.18 (0.81, 1.46)	0.182	1.18 (0.95, 1.46)	0.143
**Homicide (versus no homicide)**					
**First variable**	Poverty early adulthood	1.62 (0.05, 14.48)	0.441	1.59 (0.55, 4.58)	0.387
**Second variable**	Accumulation	1.36 (0.31, 4.48)	0.343	1.32 (0.81, 2.37)	0.236

Odds ratios (OR) and 95% confidence intervals (95% CIs) estimated from fixed Least Absolute Shrinkage and Selection Operator inference (i.e., penalized models) and Bonferroni-corrected standard logistic regression. ORs are unstandardised. All models are unadjusted.

† *P* values derived via bootstrapped confidence intervals (95% and 99%).

**PP* < .05; ***P* < .01.

Sensitivity analyses

Supplementary Table S6. Sensitivity analyses for definition of poverty (<1 BMMW) when testing life-course models of the influence of poverty on all violence in the 1982 Pelotas Birth Cohort (imputed sample, n=5660)

		Adjusted		Unadjusted	
		Type of poverty exposure	Association with outcome	Poverty Model(s) Selected	Association with outcome
			OR (95% CI)		OR (95% CI)
**Violence**					
	**First variable**	Accumulation	1.36 (1.20, 1.51)*	Accumulation	1.49 (1.33, 1.63)**
	**Second variable**	Poverty early adulthood	1.80 (1.00, 2.48)	Poverty early adulthood	1.51 (1.00, 2.03)

Odds ratios (OR) and 95% confidence intervals (95% CIs) estimated using bootstrapping on 20 multiply imputed datasets by chained equations (MICE). P values derived via bootstrapped confidence intervals (95% and 99%). Adjusted models adjust for sex, maternal age, and maternal education, which were all measured at birth. Poverty defined as <1 Brazilian monthly minimum wage (BMMW). Only all violence models could be estimated; for homicide cell counts were too low for this definition of poverty.
**P* < .05; ***PP* < .01.

Supplementary Table S7. Sensitivity analyses of poverty defined in relative terms, when testing life-course models of the influence of poverty on all violence and homicide in the 1982 Pelotas Birth Cohort (imputed sample, n=5660)

		Adjusted		Unadjusted	
		Type of poverty exposure	Association with outcome	Poverty Model(s) Selected	Association with outcome
			OR (95% CI)		OR (95% CI)
**Violence**					
	**First variable**	Accumulation	1.34 (1.21, 1.47)**	Accumulation	1.42 (1.30, 1.54)**
	**Second variable**	Poverty early adulthood	1.39 (1.14, 1.7)**	Poverty early adulthood	1.21 (1.00, 1.48)
**Homicide**					
	**First variable**	Poverty early adulthood	3.01 (1.00, 5.72)	Poverty early adulthood	2.71 (1.00, 5.01)

Odds ratios (OR) and 95% confidence intervals (95% CIs) estimated using bootstrapping on 20 multiply imputed datasets by chained equations (MICE). P values derived via bootstrapped confidence intervals (95% and 99%). Adjusted models adjust for sex, maternal age, and maternal education, which were all measured at birth. Relative poverty defined as lower income tertile at each age. SCLMA only selected one explanatory variable (poverty in early adulthood) for homicide in these models.
*$PP < .05$; **$P < .01$.

Supplementary Table S8. Sensitivity analyses for measure of violence (restricted official records and self-report) when testing life-course models of the influence of poverty on violence in the 1982 Pelotas Birth Cohort (imputed sample, n=5660)

		Adjusted		Unadjusted	
		**Type of poverty exposure**	**Association with outcome OR (95% CI)**	**Poverty Model(s) Selected**	**Association with outcome OR (95% CI)**
**Restricted violence records (>22 years)**					
	**First variable**	Accumulation	1.39 (1.21, 1.57)*	Accumulation	1.51 (1.33, 1.68)**
	**Second variable**	Poverty early adulthood	1.82 (1.00, 2.59)	Poverty early adulthood	1.54 (1.00, 2.12)
**Self-reported violence**					
	**First variable**	Poverty early adulthood	1.28 (1.00, 1.54)	Poverty early adulthood	1.49 (1.19, 1.67)*
	**Second variable**	Downward mobility (early childhood to early adulthood)	1.24 (1.00, 1.50)	Poverty at birth	1.22 (1.00, 1.37)

Odds ratios (OR) and 95% confidence intervals (95% CIs) estimated using bootstrapping on 20 multiply imputed datasets by chained equations (MICE). P values derived via bootstrapped confidence intervals (95% and 99%). Adjusted models adjust for sex, maternal age, and maternal education, which were all measured at birth.

**P* < .05; ***P* < .01.

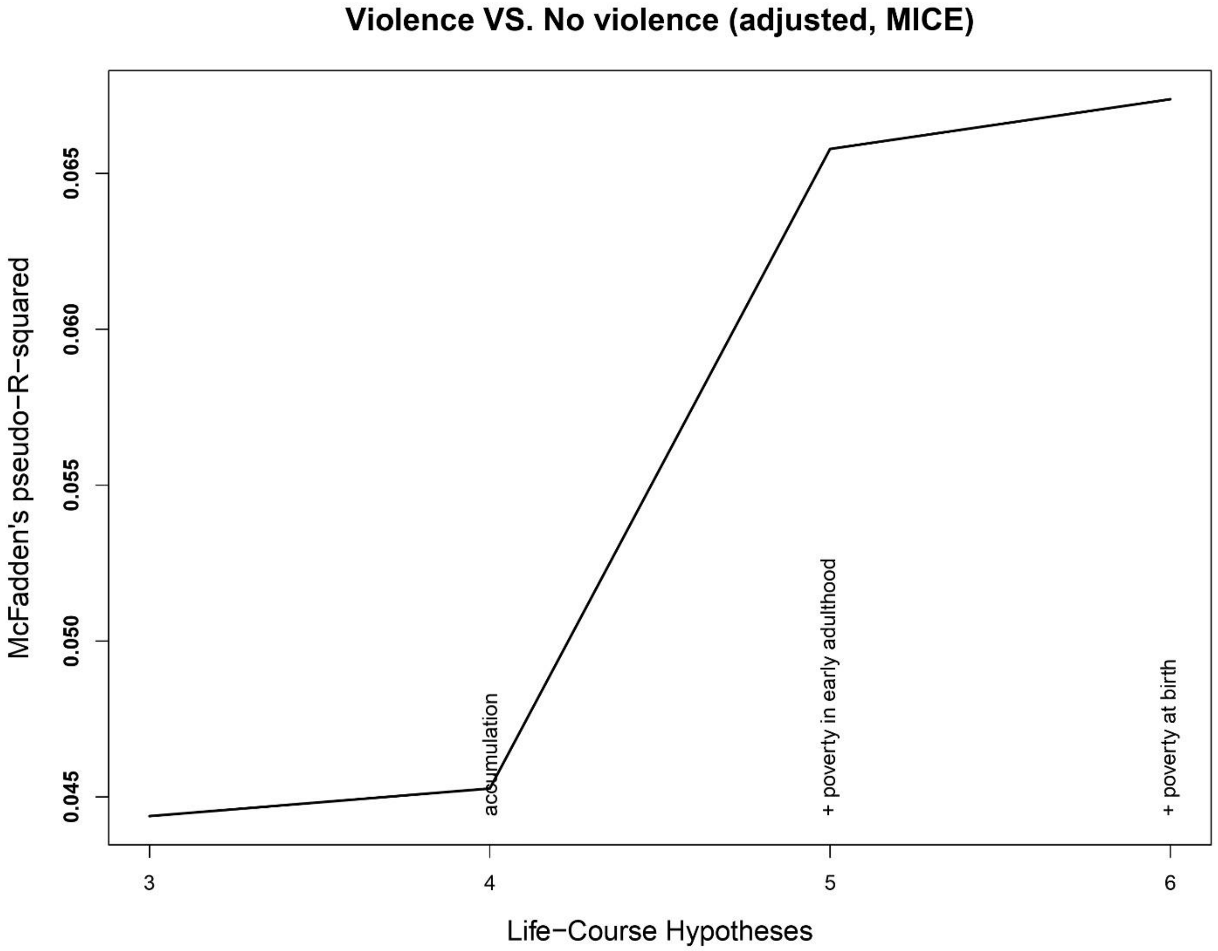

Supplementary Figure S1. Elbow plot of coefficient of variation against number of variables encoding life-course hypotheses selected at each stage, for association between life-course poverty and violent offending using multiple imputation, after adjusting for confounders (sex, maternal age, maternal education).

Note: Least Absolute Shrinkage and Selection begins by first identifying the single variable with the strongest association to the outcome; it then identifies the combination of two variables with the strongest association, followed by three variables, and so on.

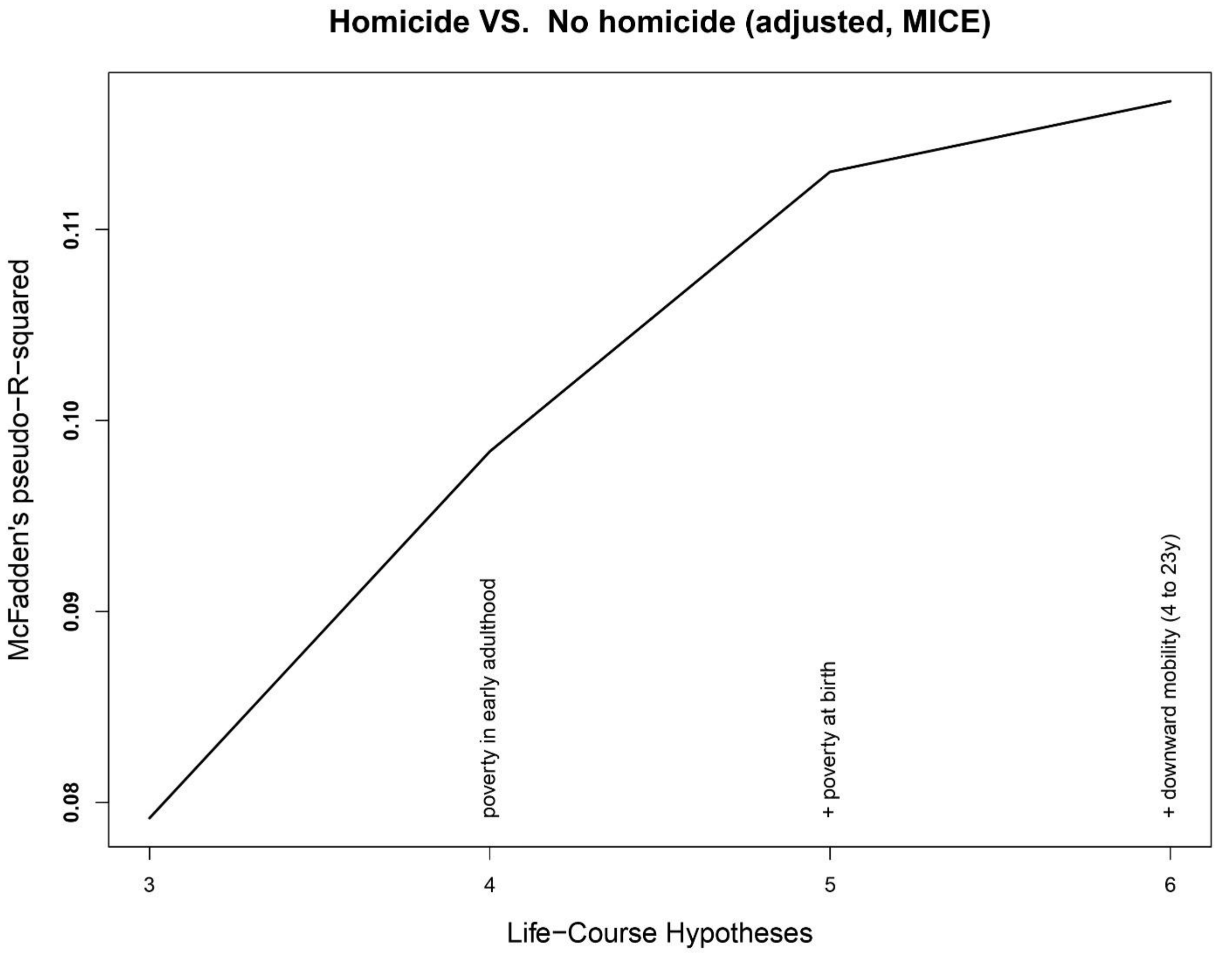

Supplementary Figure S2. Elbow plot of coefficient of variation against number of variables encoding life-course hypotheses selected at each stage using multiple imputation, for association between life-course poverty and homicide, after adjusting for confounders (sex, maternal age, maternal education).

Note: Least Absolute Shrinkage and Selection begins by first identifying the single variable with the strongest association to the outcome; it then identifies the combination of two variables with the strongest association, followed by three variables, and so on.

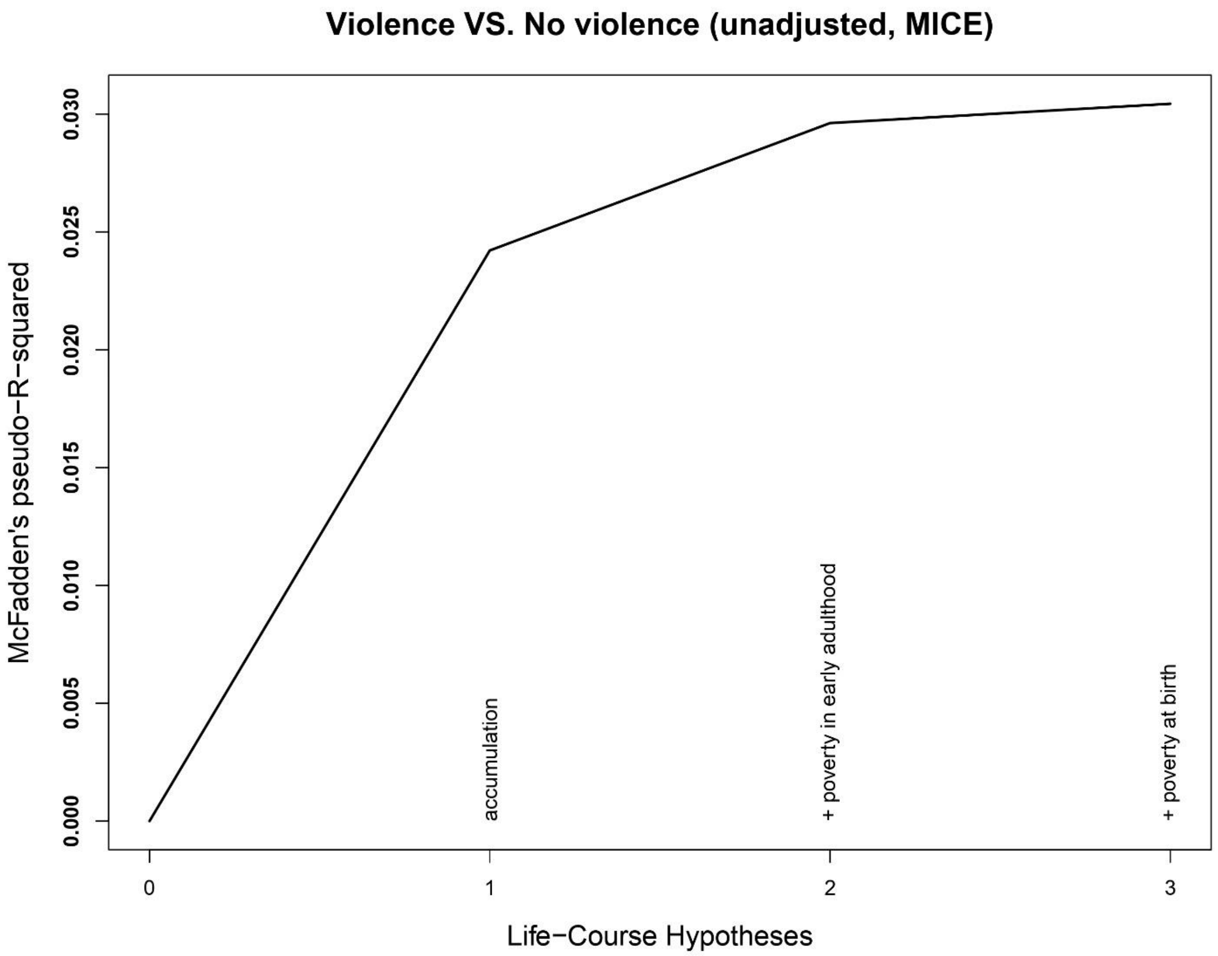

Supplementary Figure S3. Elbow plot of coefficient of variation against number of variables encoding life-course hypotheses selected at each stage using multiple imputation, for association between life-course poverty and violent offending.

Note: Least Absolute Shrinkage and Selection begins by first identifying the single variable with the strongest association to the outcome; it then identifies the combination of two variables with the strongest association, followed by three variables, and so on.

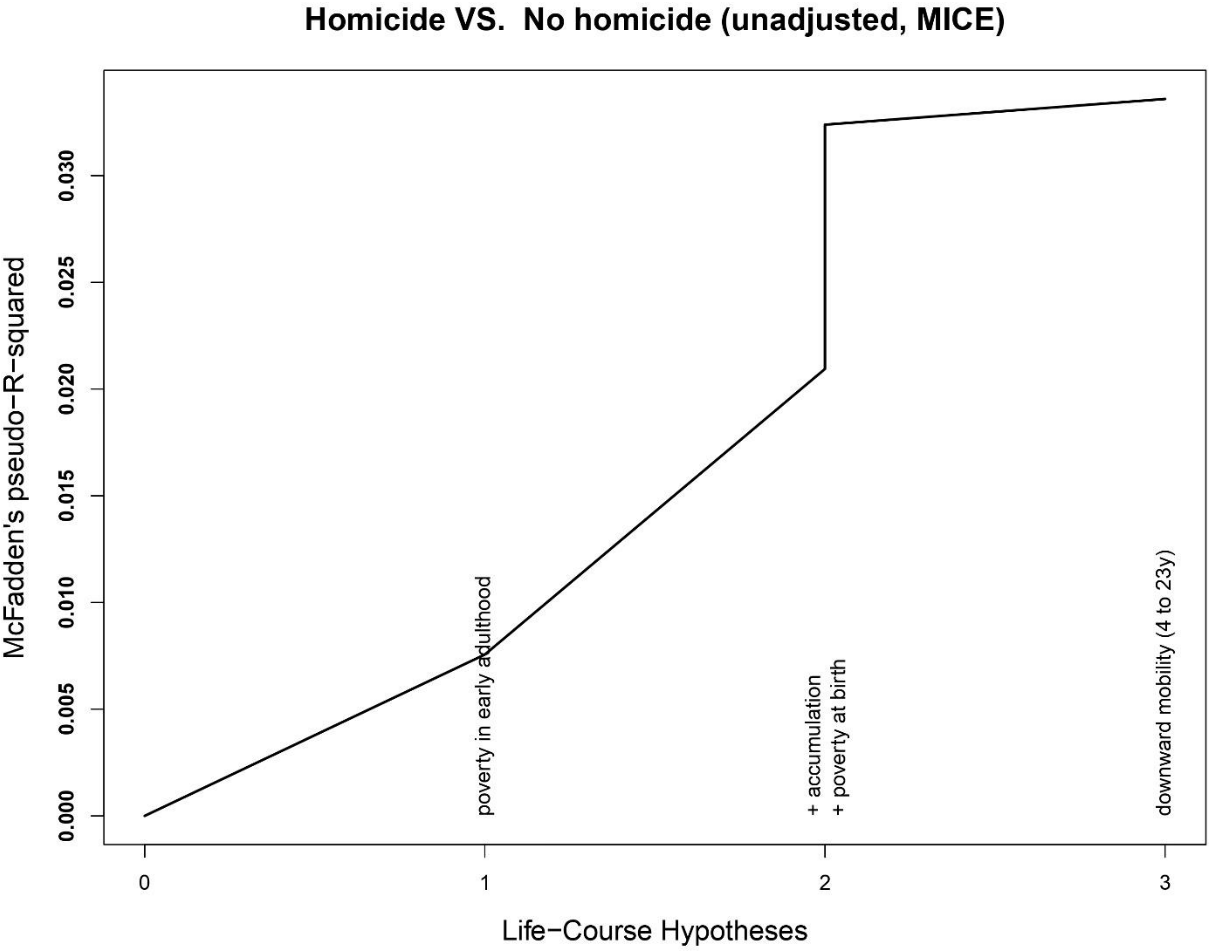

Supplementary Figure S4. Elbow plot of coefficient of variation against number of variables encoding life-course hypotheses selected at each stage using multiple imputation, for association between life-course poverty and homicide.

Note: Least Absolute Shrinkage and Selection begins by first identifying the single variable with the strongest association to the outcome; it then identifies the combination of two variables with the strongest association, followed by three variables, and so on.

Including additional variables in the model after *poverty in adulthood* resulted in unreliable estimates of additional variance explained. However, the lasso model determined that *accumulation of poverty* was the second most important explanatory variable to include, and coefficient estimates for that variable are therefore shown, in addition to poverty in adulthood, in Table 5.

References

1. Mostafa, J. and K.D. Silva, *Brazil's single registry experience: A tool for pro-poor social policies*. 2007: Brazilian Ministry of Social Development and Fight Against Hunger.
2. Little, R. and D.B. Rubin, *Statistical Analysis with Missing Data*. Vol. 333. 2014: John Wiley & Sons.
3. Nguyen, C.D., J.B. Carlin, and K.J. Lee, *Model checking in multiple imputation: an overview and case study*. Emerg Themes Epidemiol, 2017. **14**(1): p. 8.
